# Accuracy of echocardiographic estimates of pulmonary artery pressures in pulmonary hypertension: insights from the KARUM hemodynamic database

**DOI:** 10.1007/s10554-021-02315-y

**Published:** 2021-06-19

**Authors:** Ashwin Venkateshvaran, Natavan Seidova, Hande Oktay Tureli, Barbro Kjellström, Lars H. Lund, Erik Tossavainen, Per Lindquist

**Affiliations:** 1 Cardiology Unit, Department of Medicine, Karolinska Institutet, Stockholm, Sweden; 2 Department of Clinical Physiology, Danderyds Hospital, Stockholm, Sweden; 3 Department of Clinical Physiology, Surgical & Perioperative Sciences, Umeå University, Umeå, Sweden; 4 Clinical Physiology, Department of Clinical Sciences Lund, Lund University, Skåne University Hospital, Lund, Sweden; 5 Department of Cardiology, Public Health & Clinical Medicine, Umeå University, Umeå, Sweden; 6 Department of Cardiovascular Research, Karolinska University Hospital, 17176 Stockholm, Sweden

**Keywords:** Doppler echocardiography, Right heart catheterization, Tricuspid regurgitation peak velocity

## Abstract

Accurate assessment of pulmonary artery (PA) pressures is integral to diagnosis, follow-up and therapy selection in pulmonary hypertension (PH). Despite wide utilization, the accuracy of echocardiography to estimate PA pressures has been debated. We aimed to evaluate echocardiographic accuracy to estimate right heart catheterization (RHC) based PA pressures in a large, dual-centre hemodynamic database. Consecutive PH referrals that underwent comprehensive echocardiography within 3 h of clinically indicated right heart catheterization were enrolled. Subjects with absent or severe, free-flowing tricuspid regurgitation (TR) were excluded. Accuracy was defined as mean bias between echocardiographic and invasive measurements on Bland–Altman analysis for the cohort and estimate difference within ± 10 mmHg of invasive measurements for individual diagnosis. In 419 subjects, echocardiographic PA systolic and mean pressures demonstrated minimal bias with invasive measurements (+ 2.4 and + 1.9 mmHg respectively) but displayed wide limits of agreement (− 20 to + 25 and − 14 to + 18 mmHg respectively) and frequently misclassified subjects. Recommendation-based right atrial pressure (RAP) demonstrated poor precision and was falsely elevated in 32% of individual cases. Applying a fixed, median RAP to echocardiographic estimates resulted in relatively lower bias between modalities when assessing PA systolic (+ 1.4 mmHg; 95% limits of agreement + 25 to − 22 mmHg) and PA mean pressures (+ 1.4 mmHg; 95% limits of agreement + 19 to − 16 mmHg). Echocardiography accurately represents invasive PA pressures for population studies but may be misleading for individual diagnosis owing to modest precision and frequent misclassification. Recommendation-based estimates of RAP_mean_ may not necessarily contribute to greater accuracy of PA pressure estimates.

**Supplementary Information:**

The online version contains supplementary material available at 10.1007/s10554-021-02315-y.

## Introduction

Accurate hemodynamic evaluation of pulmonary hypertension (PH) is essential for early disease identification, selection for potential therapy and during follow-up. Although PH diagnosis is established using right heart catheterization (RHC), transthoracic echocardiography is recommended for screening patients [1] and routinely utilized to quantify pulmonary artery (PA) pressures in addition to offering prognostic insight [2].

The accuracy and precision of echocardiography to assess PA pressures has been debated. Multiple earlier studies suggest that echocardiographic estimates of PA pressures are frequently innacurate [3–6], while more recent publications suggest good diagnostic accuracy [7–9]. These paradoxical observations may be attributed to diverse methodological approaches to assess accuracy in the aforementioned studies [9], and compounded to some degree by varying recommendations to quantify PH using echocardiography [1, 10]. More recently, D’Alto and colleagues demonstrated high echocardiographic accuracy to estimate both PA mean (PAP_mean_) and systolic pressures (PAP_systolic_) employing Bland–Altman analysis, suggesting appropriate utility in population studies [9]. However, modest precision represented by wide limits of agreement in that study advocates greater caution when employing echocardiography to estimate PH severity on an individual basis.

Given the practicality, low cost and low risk of echocardiography, this study was undertaken to investigate its accuracy to estimate PA pressures in a large, prospective, dual-centre database of PH referrals. Further, we wished to study the contribution of mean right atrial pressure (RAP_mean_) estimates to PA pressure estimation by comparing the accuracy of the recommended approach that takes into consideration patient-specific mean right atrial mean pressure (RAP_mean_) estimates [10], and a simplified model that applies a fixed, median RAP to estimate PAP_systolic_ and PAP_mean_ in all subjects.

## Methods

### Study population

Consecutive patients with unexplained dyspnoea referred for RHC to PH referral centres at Karolinska University Hospital (2014–2018) and Norrlands University Hospital (2010–2015) were enrolled in the Karolinska-Umeå (KARUM) hemodynamic database. All subjects were hemodynamically stable during assessment and medical therapy was suitably titrated. The study was approved by the local ethics committees (Karolinska: DNR 2008/1695-31 & Norrland: 07-092 M, 2014-198-32 M,2017-102-32 M). All patients provided written informed consent.

### Echocardiographic evaluation

All patients underwent comprehensive echocardiography within 3 h of catheterization at both centers employing a Vivid E9 ultrasound system (GE Ultrasound, Horten, Norway) in keeping with current recommendations [10]. Pharmacological status was unaltered between echocardiography and RHC examinations. All studies were performed by credentialed echocardiographers with > 15 years’ experience at each center (PL/AV). 2D gray-scale images were acquired at 50–80 frames/sec and Doppler tracings were recorded using a sweep speed of 100 mm/sec. Three consecutive heart cycles were acquired in sinus rhythm and 5 in the setting of atrial fibrillation (AF). TRV_max_ was measured with Continuous wave Doppler, considering the most optimal signal obtained from multiple echocardiographic windows. RAP_mean_ was estimated by evaluating inferior vena cava (IVC) size and collapsibility with patients in a supine position, taking care to maximize IVC diameter both during relaxed respiration and with rapid inspiration. All images were subsequently exported and analyzed offline (EchoPAC PC, version 11.0.0.0 GE Ultrasound, Waukesha, Wisconsin) by experienced investigators (PL/AV) blinded to catheterization data.

Subjects with absent or poor TR signal quality, in addition to those with a flail tricuspid valve, endocarditis or a coaptation defect resulting in massive, free-flowing jet were subsequently excluded from the analysis. TR severity was assessed semi-quantitatively and graded as mild (grade 1), moderate (grade 2) and moderately-severe to severe (grade 3). In keeping with American Society of Echocardiography/European Association of Cardiovascular Imaging (ASE/EACVI) recommendations, RAP_mean_ was estimated as 3 mmHg if the IVC diameter was < 2.1 cm and collapsed > 50% during rapid inspiration and 15 mmHg if the IVC diameter was ≥ 2.1 cm and collapsed < 50%. In scenarios where IVC size and dynamics did not fit this paradigm (IVC diameter < 2.1 cm with < 50% collapse and IVC diameter ≥ 2.1 cm with > 50% collapse), RAP_mean_ was estimated as 8 mmHg [10]. In the simplified model, median RAP was uniformly applied to corresponding TRV_max_ gradients to estimate PAP_systolic_. PAP_mean_ was calculated as 0.6 × PAP_systolic_ + 2 [11] using both recommended [10] and fixed, median RAP estimates.

### Invasive evaluation

RHC was performed by experienced operators blinded to echocardiography examinations at each center using a 6F Swan Ganz catheter employing jugular or femoral vein access. After suitable calibration with the zero-level set at the mid-thoracic line, pressure measurements were taken from the right atrium (RA), right ventricle (RV) and PA during end-expiration. Five to ten cardiac cycles were acquired and all pressure tracings were stored and analyzed offline using a standard hemodynamic software package (WITT Series III, Witt Biomedical Corp., Melbourne, FL).

### Statistical analysis

Normality was tested using the Shapiro–Wilk test and visually reaffirmed using QQ plots. Continuous variables were expressed as mean ± SD for parametric variables or median (interquartile range) for non-parametric variables and categorical variables were expressed as numbers and percentage. Correlations between echocardiographic and corresponding invasive measurements was performed using the Pearson’s 2-tailed test (correlation between 2 continuous variables). Receiver operating characteristics (ROC) curve was employed to illustrate diagnostic potential of each chosen variable. An invasive PA mean pressure (PAP_mean_) ≥ 25 mmHg was chosen to represent PH and RAP_mean_ ≥ 7 mmHg, to represent an elevated RAP_mean_ [12]. Sensitivity, specificity, negative predictive value (NPV) and positive predictive value (PPV) were measured. Accuracy was assessed both for individual diagnosis and at the cohort level. Accuracy for individual diagnosis was predefined as an estimate difference within ± 10 mmHg of invasive measurements. Accuracy at the cohort level was defined as the mean bias between echocardiographic and invasive measurements on Bland–Altman analysis. IBM SPSS statistics version 23.0 was employed for analysis.

## Results

### Study population

**Fig. 1:**
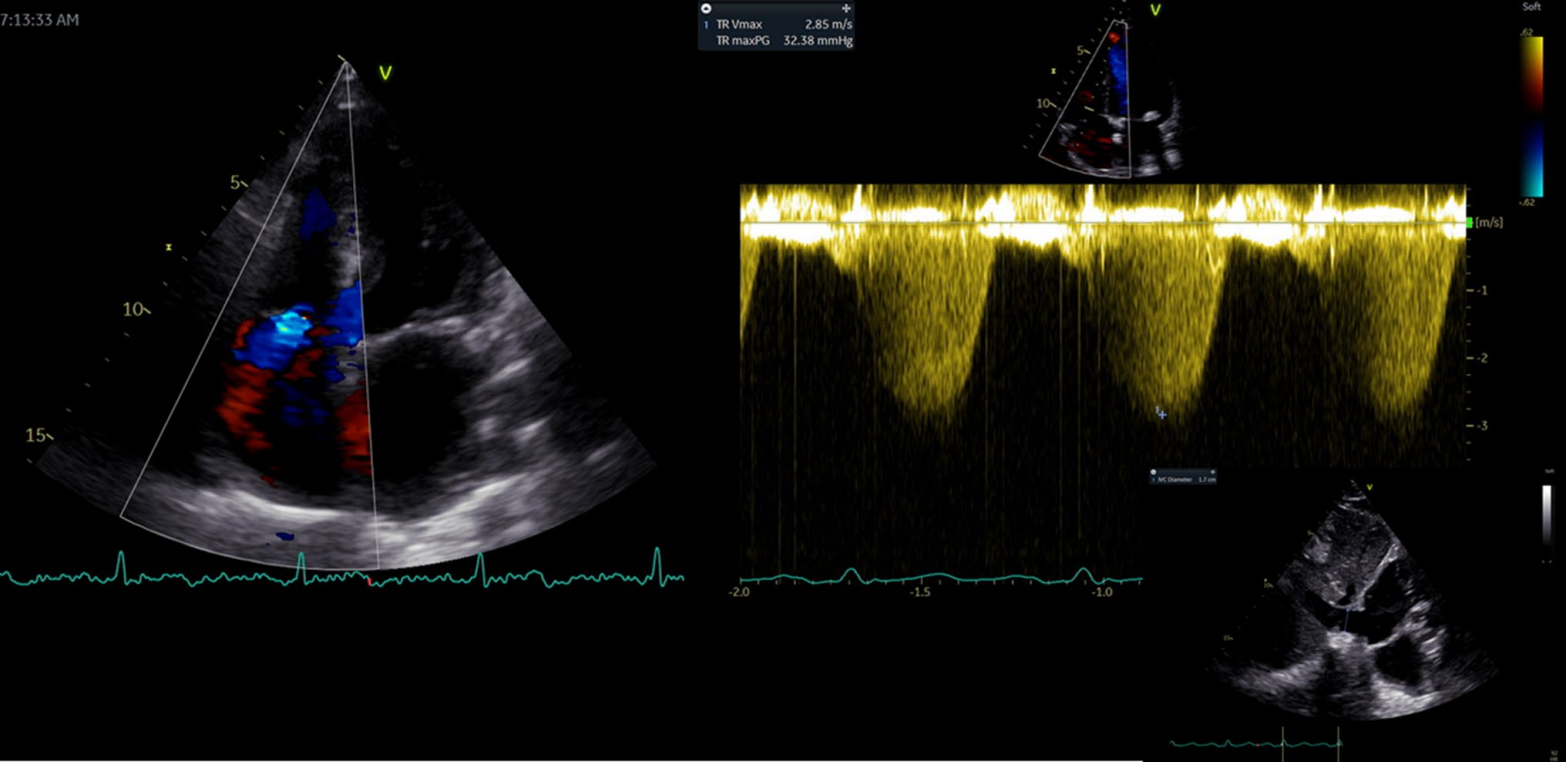
Echocardiographic pulmonary artery systolic pressure (PAP_systolic_) obtained by adding gradient corresponding with tricuspid regurgitation peak velocity to estimated right atrial pressure obtained from inferior vena cava size and collapse. Pulmonary artery mean pressure was calculated as 0.6 × PAP_systolic_ + 2

Of 480 subjects enrolled across the two sites, 47 (10%) patients with no TR and 14 (3%) with a coaptation defect resulting in severe, free-flowing TR were excluded, yielding 419 patients [Karolinska: n = 296 (70%); Umeå: n = 123 (30%)] for analysis. Clinical characteristics, invasive and echocardiographic data are provided in Table 1. Fifty-two percent of the subjects were female. Twenty percent (n = 86) presented with AF and 7% (n = 31) were on pacemaker therapy. A wide range of invasive pressures were observed for RAP_mean_ (1–29 mmHg), PAP_mean_ (7–99 mmHg) and PAP_systolic_ (12–136 mmHg). One hundred and seventy-nine patients (44%) presented with reduced RV systolic function as suggested by TAPSE < 16 mm [10]. Echocardiographic images of the IVC were either not available or did not permit optimal evaluation in a small fraction (n = 32; 7.6%). Two hundred and forty patients (57%) presented with mild TR, 122 (29%) with moderate TR, and 57 (14%) with severe TR. An illustration of echocardiographic evaluation of PA pressures is provided in Fig. 1.

**Table 1:** Clinical Characteristics, invasive and echocardiographic data of patient population. Data presented as mean ± SD/ median (Q1; Q3) or number (%)

	Patient population (n = 419)
Demographics	
Age (years)	62 ± 15
Female	218 (52)
Medical history	
Diabetes	59 (14)
Hypertension	188 (44)
Atrial fibrillation	86 (20)
Clinical assessment	
Heart rate (bpm)	72 ± 14
Body surface area (m^2^)	1.9 ± 0.9
Systolic blood pressure (mmHg)	123 ± 23
Diastolic blood pressure (mmHg)	70 ± 13
Indication for RHC	
PAH or CTEPH	169 (40)
Heart failure	176 (42)
Post-cardiac transplantation	8 (2)
Constriction	26 (6)
Arrhythmogenic right ventricular dysplasia	25 (6)
Others	15 (4)
RHC	
PAP_systolic_ (mmHg)	49 (37;66)
PAP_diastolic_ (mmHg)	20 (14;25)
PAP_mean_ (mmHg)	32 (23;41)
RAP_mean_ (mmHg)	7 (4;11)
Echocardiography	
RVID_basal_ (mm)	42 ± 8
TAPSE (mm)	17 ± 5
RA area (cm^2^)	22 ± 7
Doppler	
TRV_max_ (m/s)	3.2 (2.7;3.8)

*RHC* right heart catheterization, *PAH* pulmonary arterial hypertension, *CTEPH* chronic thromboembolic pulmonary hypertension, *PAP* pulmonary artery pressure, *RAP* right atrial pressure, *RVID* right ventricular internal diameter end-diastole, *TAPSE* tricuspid annular plane systolic excursion, *TRV*_*max*_ tricuspid regurgitation max velocity, *RA* right area

### Accuracy of TRV_max_ to identify presence of pH

TRV_max_ demonstrated strong association with invasive PAP_mean_ (r = 0.75, p < 0.001) and a cut-off of 2.8 m/sec demonstrated good ability to identify PH, defined as invasive PAP_mean_ ≥ 25 mmHg (AUC = 0.87, CI 0.84–0.91, p < 0.001). Sensitivity analysis for different echocardiographic cut-offs to is presented in Table 2. At 2.8 m/sec, TRV_max_ demonstrated 89% sensitivity and 62% specificity to identify PH, with a 38% false positive rate. Forty-five patients (15%) with a TRV_max_ > 2.8 m/sec demonstrated normal PA pressures on RHC. At 3.4 m/sec, TRV_max_ demonstrated 94% specificity and 62% sensitivity, and false positive rate fell to 5.9%. Even when balanced sensitivity and specificity was identified at a 3.0 m/sec cut-off (80% sensitivity, 80% specificity), a 20% false positive rate was observed. Supplementary sensitivity analysis was also performed considering PAP_mean_ ≥ 20 mmHg which revealed similar results (Online Appendix Table 1). On Bland–Altman analysis, echocardiographic TR gradient demonstrated a mean bias of + 2.5 mmHg with invasive RV-RA gradient (95% limits of agreement + 23 to − 18 mmHg).

**Table 2:** Sensitivity, specificity, positive predictive value, negative predictive value for echocardiographic cut-offs to identify corresponding RHC values

	Cut off	RHC value	Sensitivity (%)	Specificity (%)	Positive predictive value (%)	Negative predictive value (%)
TRV_max_	2.8 m/sec	PAP_mean_ ≥ 25 mmHg	89	62	85	68
TRV_max_	3.0 m/sec	PAP_mean_ ≥ 25 mmHg	80	80	90	62
TRV_max_	3.4 m/sec	PAP_mean_ ≥ 25 mmHg	62	94	96	50
Estimated RAP	7 mmHg	RAP_mean_ > 7 mmHg	84	68	69	85

### Accuracy of IVC to estimate RAP_mean_ categories

RAP_mean_ estimated as per ASE/EACVI recommendations [10] demonstrated a good ability to identify invasive RAP_mean_ > 7 mmHg (AUC: 0.80; CI 0.76–0.85, p < 0.001). However, Sensitivity analysis demonstrated a modest 68% specificity and 69% PPV (Table 2). Further, 67 subjects (32%) that demonstrated elevated RAP_mean_ estimated by echocardiography demonstrated normal invasive RAP_mean_. When invasive RAP_mean_ was plotted against echocardiographic estimates, median (IQR) for the 3, 8 and 15 mmHg IVC-estimated subgroups were 5 (3–7 mmHg), 8 (5–10 mmHg) and 13 (8–16 mmHg) (p < 0.001 for comparison between groups). A total of 122 patients displayed an IVC-estimated RAP_mean_ of 15 mmHg. In this subgroup, 15 (12%) demonstrated an invasive RAP_mean_ < 5 mmHg, and 45 (37%), an RAP_mean_ ≤ 10 mmHg. On Bland–Altman analysis, minimal bias but poor precision was observed between modalities (mean bias: − 0.1 mmHg; 95% limits of agreement + 9.1 to − 9.5 mmHg).

### Accuracy of echocardiography to evaluate invasive PAP_systolic_

**Fig. 2:**
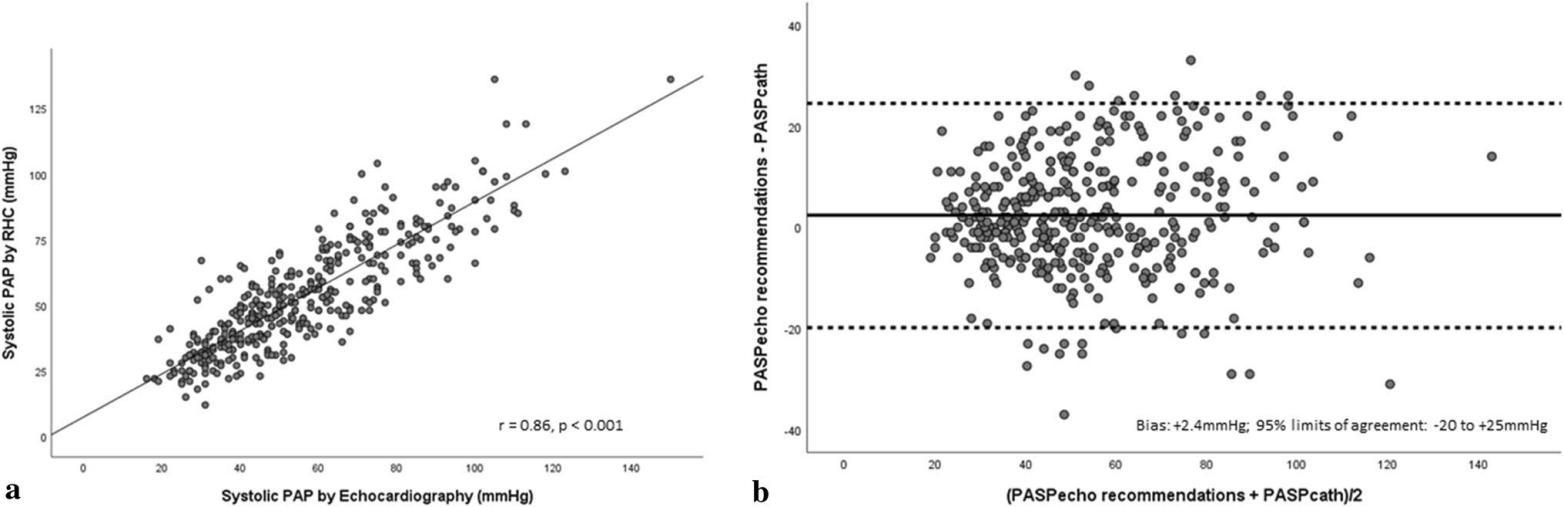
**a** Scatter plot demonstrating correlation between PAP_systolic_ estimated by echocardiography employing current ASE/EACVI recommendations and RHC. **b** Bland–Altman plot demonstrating agreement between PAP_systolic_ estimated by estimated by echocardiography employing current ASE/EACVI recommendations and RHC

**Fig. 3:**
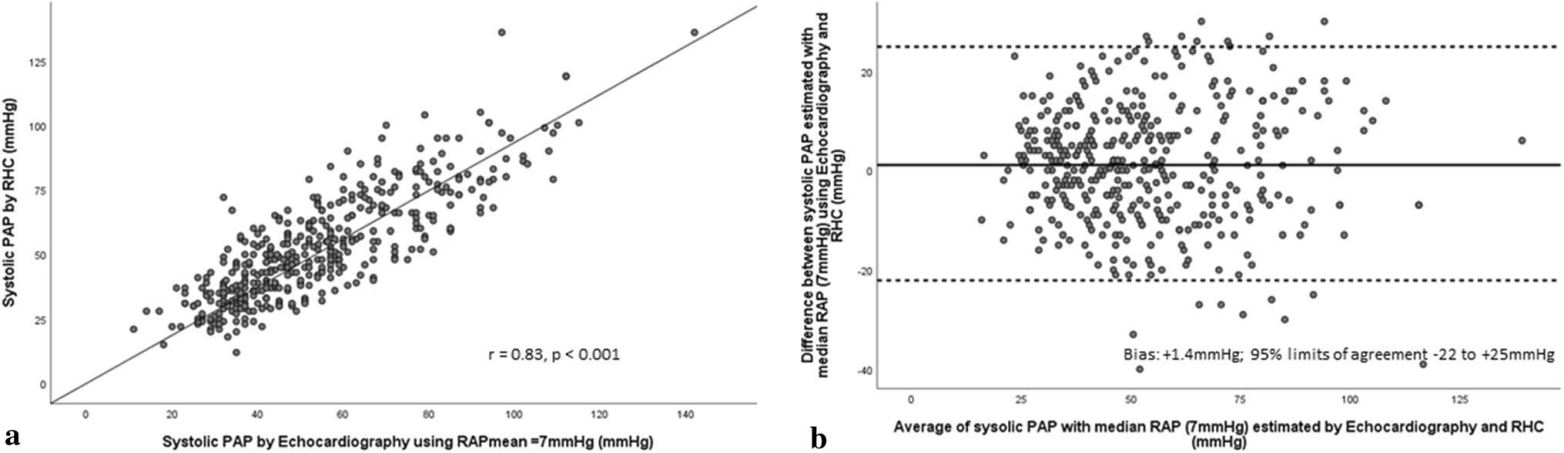
**a** Scatter plot demonstrating correlation between PAP_systolic_ estimated by echocardiography using RAP = 7 mmHg and RHC. **b** Bland–Altman plot demonstrating agreement between PAP_systolic_ estimated by estimated by echocardiography using RAP = 7 mmHg and RHC

Echocardiographic PAP_systolic_ as per the ASE/EACVI approach demonstrated strong association with invasive PAP_systolic_ (r = 0.86, p < 0.001) (Fig. 2a). Bias and limits of agreement between echocardiographic estimates of PAP_systolic and_ RHC are presented in Table 3 and Fig. 2b. Bland–Altman analysis revealed low bias between echocardiography and RHC (mean bias =  + 2.4 mmHg; CI 1.2–3.5 mmHg) with wide limits of agreement (− 20 to + 25 mmHg) (Fig. 2b). Only 62% of individual echocardiographic estimates were accurate. Echocardiography overestimated RHC by > 10 mmHg in 92 of 387 estimates (24%) and underestimated RHC by > 10 mmHg in 36 of 387 estimates (10%). Absolute values for magnitude of overestimation were comparable with underestimation (18 ± 5 vs. 18 ± 6 mm). When median RAP_mean_ (7 mmHg) was incorporated instead of IVC-based estimates [10], association between echocardiographic and invasive PAP_systolic_ remained strong (r = 0.83, p < 0.001) (Fig. 3a). Bland–Altman analysis displayed relatively lower mean bias between methods (Bias: + 1.4 mmHg, 95%CI 0.2–2.5 mmHg) and comparable limits of agreement (− 22 to + 25 mmHg) when compared with the ASE/EACVI approach (Fig. 3b).

**Table 3:** Bias and limits of agreement between echocardiographic estimates of systolic and mean pulmonary artery pressures and right heart catheterization

Echo estimate	Bias ± SD	95% CI	Lower limit (Mean-2SD)	Upper limit (Mean + 2SD)
PAP_systolic (ASE/EACVI) (mmHg)_	+ 2.4 ± 11	1.2–3.5	− 20	+ 25
PAP_systolic (RAP = 7 mmHg) (mmHg)_	+ 1.4 ± 12	0.2–2.5	− 22	+ 25
PAP_mean (ASE/EACVI) (mmHg)_	+ 1.9 ± 8	1.0–2.6	− 14	+ 18
PAP_mean (RAP = 7 mmHg) (mmHg)_	+ 1.4 ± 9	0.5–2.2	− 16	+ 19

### Accuracy of echocardiography to evaluate invasive PAP_mean_

**Fig. 4:**
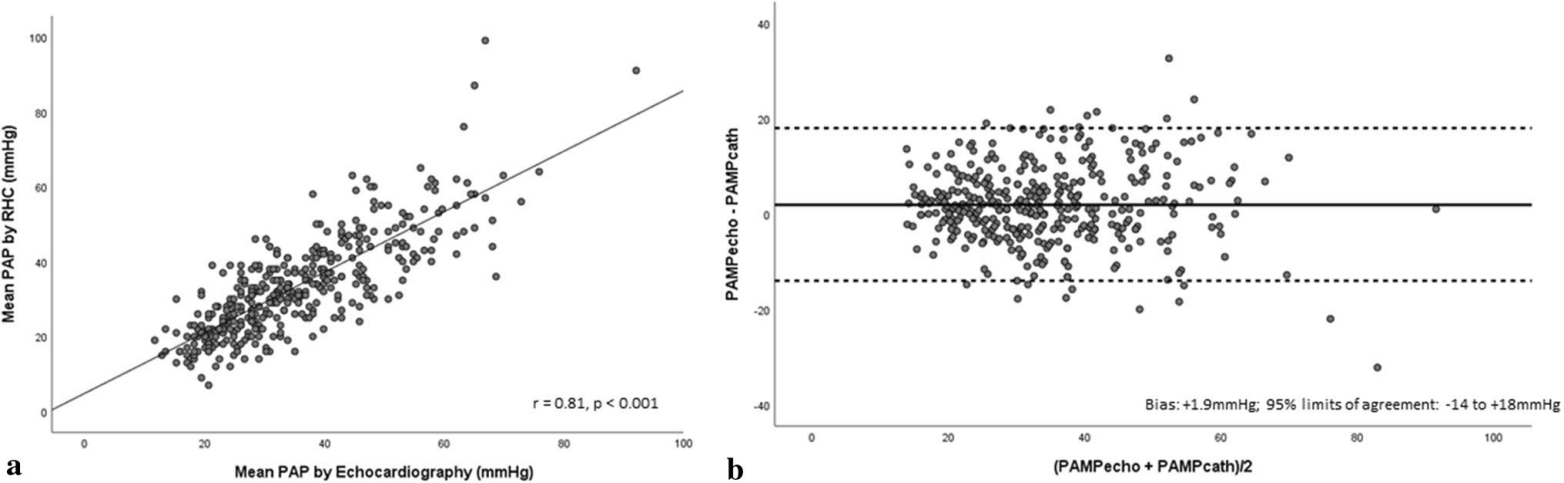
**a** Scatter plot demonstrating correlation between PAP_mean_ estimated by echocardiography employing current ASE/EACVI recommendations and RHC. **b** Bland–Altman plot demonstrating agreement between PAP_mean_ estimated by echocardiography employing current ASE/EACVI recommendations and RHC

**Fig. 5:**
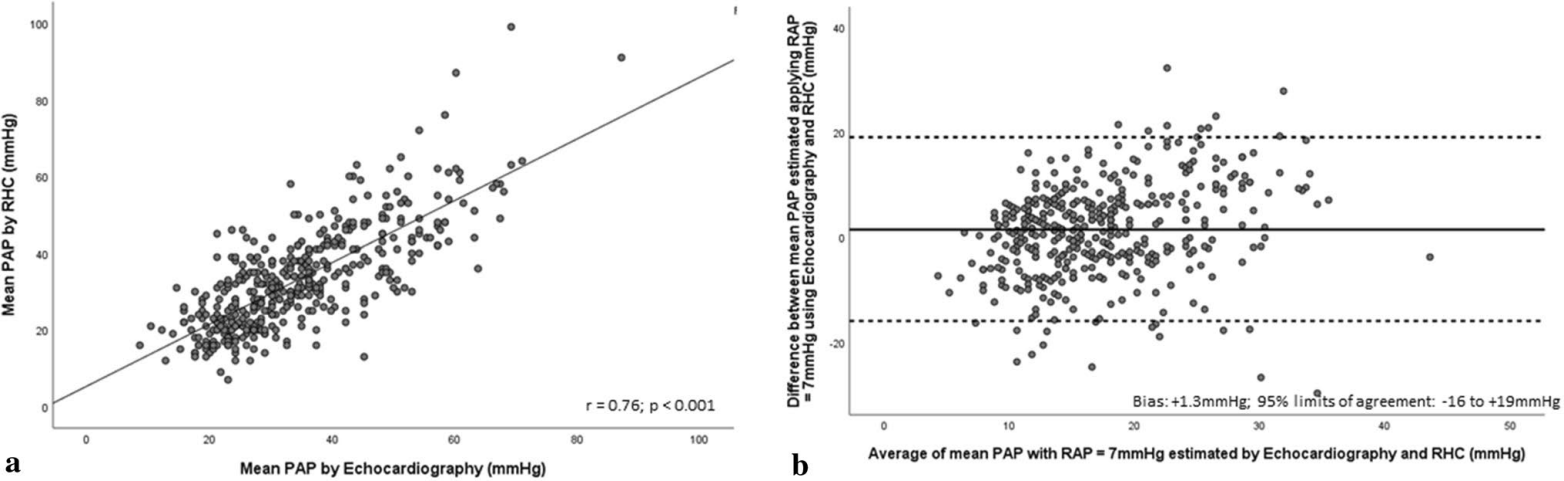
**a** Scatter plot demonstrating correlation between PAP_mean_ estimated by echocardiography employing RAP = 7 mmHg and RHC. **b** Bland–Altman plot demonstrating agreement between PAP_mean_ estimated by echocardiography employing RAP = 7 mmHg and RHC

Echocardiographic PAP_mean_ incorporating recommendation-based RAP estimates [10] demonstrated strong association with invasive PAP_mean_ (r = 0.81, p < 0.001) (Fig. 4a). Bias and limits of agreement between echocardiographic estimates of PAP_mean and_ RHC are presented in Table 3. Bland–Altman analysis revealed low bias between methods (mean bias =  + 1.9 mmHg; 95%CI 1.0–2.6 mmHg) with modest precision (limits of agreement = − 14 to + 18 mmHg) (Fig. 4b). Applying an RAP_mean_ = 7 mmHg to echocardiographic PAP_mean_ lowered bias between methods (mean bias =  + 1.3 mmHg; 95%CI 0.5–2.2 mmHg) and showcased comparable limits of agreement (− 16 to + 19 mmHg) (Fig. 5b).

## Discussion

In the large, dual-centre KARUM hemodynamic database, echocardiographic PA pressures were reasonably accurate, demonstrating strong association and minimal bias with corresponding pressures obtained by RHC. However, wide limits of agreement in addition to frequent misclassification suggests modest precision and precludes wider echocardiographic utilization to quantify PH severity in individual cases. An important observation was that recommended echocardiographic estimates of RAP_mean_ were falsely elevated in more than 1 in 3 subjects and incorporation of these estimates to calculate PAP_systolic_ and PAP_mean_ resulted in relatively higher mean bias with RHC than when the median estimate was considered for all subjects.

Interest in the utility of echocardiography to estimate PA pressures emerged with early studies suggesting a significant correlation between TR-derived estimates and invasive pulmonary pressures [13–15]. Since then, estimation of TRV_max_ has been routinely utilized to grade PH probability [1, 16] and to derive PA systolic pressures using the Bernoulli equation. In keeping with earlier studies, we demonstrate that despite a significant correlation between invasive and echocardiographic measurements, frequent misclassifications may occur when individual cases are considered [3, 4]. A number of reasons have previously been proposed to explain poor accuracy in the setting of specific cases, and these have been considered and explored in our work. First, poor agreement with invasive pressures has been previously documented in subjects with absent [17] and severe, free-flowing TR secondary to a coaptation defect [18]. Both these groups were excluded from our analysis to circumvent any bias or negative influence on our results. Second, application of the Bernoulli equation to TR velocity to calculate pressure gradient is inherently error-prone, as even small errors in absolute measurement result in exponential differences in PAP_systolic_ estimates. Certain international recommendations hence encourage the use of absolute velocities instead [1, 16]. Our data shows that the recommended 2.8 m/sec cut-off for intermediate-probability PH misclassified one in three subjects and raising the cut-off to 3.4 m/sec resulted in a drop in sensitivity, thereby warranting re-evaluation of these recommended values. Finally, the integration of IVC-derived RAP estimates to corresponding TR gradients is recommended to calculate PAP_systolic_ [10]_._ The reliability of this method to represent invasive RAP_mean_ has been debated [19–24], with certain studies suggesting modest or no association [20, 21, 24]. While IVC-estimated RAP demonstrated good ability to identify elevated invasive RAP_mean_ in our study, accuracy of recommended cut-offs to represent invasive pressures was poor and may explain the frequent misrepresentation of pulmonary systolic pressures. When a fixed median RAP of 7 mmHg was considered in the population, association was still strong and bias between echocardiographic and invasive PAP_systolic_ and PAP_mean_ readings was actually lower in our study, suggesting that these recommended estimates may offer no additional advantage to PA pressure assessment [25]. A recent study suggests that echocardiography frequently underestimates PA pressures owing to the inability to accurately assess elevated RA pressures during exercise [7]. Our findings suggest that the echocardiographic assessment of RAP_mean_ is frequently inaccurate even during rest, and results in frequent overestimation of invasive pressures. Objective assessment of the IVC demonstrates inherent technical limitations related to excessive translation during rapid inspiration [26] and has been previously reported to be inaccurate in athletes [27] and patients on mechanical ventilation [24]. Recent studies suggest that advanced techniques such as speckle-tracking based right atrial reservoir strain [28] and 3D volumes [29] may provide a more accurate measure of RAP_mean_, but these findings require further validation in larger cohorts.

Our study also corroborates earlier findings that suggest modest echocardiographic precision to reflect invasive PA pressures when individual cases are concerned [3, 4, 9, 30], but an appropriate method for population studies given its high accuracy at a cohort level [9]. Echocardiography remains a practical, inexpensive and safe screening tool to arouse suspicion of PH, offers additional etiological insight in addition to complementary information on ventricular structural and/or functional aberrations. Further, aggravations in TR severity assessed by Doppler have been associated with worsening prognosis irrespective of PA pressures and right heart failure [31]. From a clinical stand point, our study suggests that echocardiography is useful to raise suspicion of PH and accurately represents invasive PA pressures for population studies, but sole reliance to quantify PA pressure elevations for individual diagnosis may be frequently inaccurate. A diagnostic algorithm that combines hemodynamic information with structural indices of right-heart structure and function may vastly improve accuracy of non-invasive PH estimation for individual cases and needs to be explored.

The use of fluid-filled catheters instead of high-fidelity manometer-tipped catheters for pressure measurement might introduce additional error and may be considered a limitation in this study. Additionally, we did not adopt a core lab approach to evaluation of echocardiographic images in this study and inter-operator and inter-evaluator variability may be considered a limitation. However, a standard international acquisition and analysis protocol was followed by two experienced echocardiographers with over 15 years’ experience. Finally, we did not employ agitated saline to boost weak TR signals in this study, but chose instead to exclude unanalyzable signal registrations from the analysis. Fewer cases may have been excluded with contrast use and this may be considered a limitation.

## Conclusions

Echocardiography accurately represents invasive PA pressures and is, hence, appropriate for population studies. However, modest precision and frequent misclassification preclude its utility for evaluation of PH severity in individual cases. Recommendation-based estimates of RAP_mean_ frequently overestimate corresponding invasive measurements and do not necessarily contribute to greater accuracy of pulmonary artery (PA) pressure estimates.

## Supplementary Information

Below is the link to the electronic supplementary material.

Supplementary file1 (DOC 34 kb)

## Data Availability

Data that support the findings of this study are available from the corresponding author (AV) upon reasonable request.
