# Supplementary material for: Accuracy of echocardiographic estimates of pulmonary artery pressures in pulmonary hypertension: insights from the KARUM hemodynamic database
Source: Int J Cardiovasc Imaging. 2021 Jun 19;37(9):2637–45. doi: 10.1007/s10554-021-02315-y (PMC8390416; doi:10.1007/s10554-021-02315-y)
Supplement: Supplementary file 1 — Supplementary file1 (DOC 34 kb) [file 10554_2021_2315_MOESM1_ESM.doc]

**Appendix Table 1.** Sensitivity, specificity, positive predictive value, negative predictive value for echocardiographic cut-offs to identify PAP_mean_≥20mmHg by RHC

| **Echo Estimate** | **Cut off** | **RHC value** | **Sensitivity (%)** | **Specificity**  **(%)** | **Positive predictive value (%)** | **Negative predictive value (%)** |
| --- | --- | --- | --- | --- | --- | --- |
| TRV_max_ | 2.8m/sec | PAP_mean_≥20mmHg | 83 | 64 | 92 | 44 |
| TRV_max_ | 3.0m/sec | PAP_mean_≥20mmHg | 73 | 82 | 95 | 39 |
| TRV_max_ | 3.4m/sec | PAP_mean_≥20mmHg | 55 | 96 | 98 | 31 |
| PAP_systolic (ASE/EACVI)_ | 40mmHg | PAP_mean_≥20mmHg | 81 | 75 | 94 | 44 |
| PAP_systolic_  _(RAP = 7mmHg)_ | 40mmHg | PAP_mean_≥20mmHg | 79 | 71 | 93 | 43 |

RHC right heart catheterization, TRV_max_ tricuspid regurgitation peak velocity, PAP pulmonary artery pressure, RAP right atrial pressure, ASE American Society of Echocardiography, EACVI European Association of Cardiovascular Imaging
